# Foaming Betadine Spray as a potential agent for non-labor-intensive preoperative surgical site preparation

**DOI:** 10.1186/s12941-015-0076-2

**Published:** 2015-04-02

**Authors:** Roli Kargupta, Garret J Hull, Kyle D Rood, James Galloway, Clinton F Matthews, Paul S Dale, Shramik Sengupta

**Affiliations:** Department of Bioengineering, University of Missouri, 252 Agricultural Engineering Building, Columbia, MO 65211 USA; Department of Surgery, University Hospital, Columbia, MO USA

**Keywords:** Surgical site preparation, Povidone-iodine aerosol foam, Wet skin scrub Prep, Betadine, Foaming Betadine Spray

## Abstract

**Background:**

The Centers for Disease Control and Prevention’s (CDC) National Healthcare Safety Network (NHSN) report published in 2009 shows that there were about 16,000 cases of surgical site infection (SSI) following ~ 850,000 operative procedures making SSI one of the most predominant infection amongst nosocomial infections. Preoperative skin preparation is a standard procedure utilized to prevent SSIs thereby improving patient outcomes and controlling associated healthcare costs. Multiple techniques/ products have been used for pre-operative skin preparation, like 2 step scrubbing and painting, 2 step scrubbing and drying, and 1 step painting with a drying time. However, currently used products require strict, time consuming and labor-intensive protocols that involve repeated mechanical scrubbing. It can be speculated that a product requiring a more facile protocol will increase compliance, thus promoting a reduction in SSIs. Hence, the antimicrobial efficacy of a spray-on foaming formulation containing Betadine (povidone-iodine aerosol foam) that can be administered with minimum effort is compared to that of an existing formulation/technique (Wet Skin Scrub).

**Methods:**

In vitro antimicrobial activities of (a) 5% Betadine delivered in aerosolized foam, (b) Wet Skin Scrub Prep Tray and (c) liquid Betadine are tested against three clinically representative microorganisms (*S. aureus, S. epidermidis* and *P. aeruginosa,*) on two surfaces (agar-gel on petri-dish and porcine skin). The log reduction/growth of the bacteria in each case is noted and ANOVA statistical analysis is used to establish the effectiveness of the antimicrobial agents, and compare their relative efficacies.

**Results:**

With agar gel as the substrate, no growth of bacteria is observed for all the three formulations. With porcine skin as the substrate, the spray-on foam’s performance was not statistically different from that of the Wet Skin Scrub Prep technique for the microorganisms tested.

**Conclusions:**

The povidone-iodine aerosolized foam could potentially serve as a non-labor intensive antimicrobial agent for surgical site preparation.

## Background

Surgical site infection (SSI) is one of the major concerns associated with surgery [1,2]. SSIs can lead to severe complications, and also to patient mortality in extreme cases [3]. A 2009 estimate of the annual direct medical costs of Hospital Acquired Infections (HAI) by the Centers for Disease Control and Prevention (CDC) estimated the number of SSIs in USA to be >290,000 per year, with an estimated cost of resulting inpatient services of up to $ 10 Billion [4]. A reduction in the incidences of SSI would thus greatly improve patient wellbeing with a significant reduction in healthcare expenses [5,6].

Colonizing microorganisms often cause SSI. Microorganisms present on the skin and the operation room can readily enter a patient’s body through the site of an incision [7]. Transient bacteria present on the skin can and do cause SSIs, and reducing their number is of vital importance. This is achieved using a combination of processes: sterilization of the operating room, cleaning, showering and de-hairing of the surgical site, and scrubbing the surgical site with antimicrobial agents [7,8].

Commonly used antimicrobial agents are alcohol, iodine, chlorohexidine and povidone-iodine solution (PVP-I) among others [3]. They can be administered in the form of liquids and solutions [1]. The choice of antimicrobial agent and the technique of preoperative skin preparation to be used depends on various factors like surgeon’s familiarity with the technique, cost, efficacy of the agent used, ease of use, surgical site and possible bacteria present at the site [9]. There are several techniques like 1-step paint and dry method (taking about 3 minutes) or only scrub and dry techniques (taking greater than 5 minutes) or scrub-paint technique (taking about 5 minutes) [10-13]. We selected the wet skin scrub prep technique as it is a commonly used technique in the operating room, and the technique of choice for a number of clinicians at our institution. Drawbacks of the current protocols of surgical site preparation techniques are; they are laborious, time consuming, expensive and often require the user to follow certain strict protocols [3,8,14]. It is speculated that a product sprayed directly at and/or near the surgical site would be able to overcome these disadvantages, resulting in reduction in time, effort and personnel needed for surgical site preparation, and hence cost incurred.

While there are multiple products available for surgical site preparation (with varying, but broadly comparable efficacies) a common preoperative surgical site preparation technique involves a 5 minute scrub with povidone-iodine detergent [15]. If the 5-minute scrub is replaced with a spray-on protocol taking 2 minutes, it could result in saving 3 minutes during every surgical procedure. Given average operating room charges of $62/minute, this three-minute reduction in surgical site preparation will result in savings of $186/case if the cost of the spray on product is similar to the existing technique [16]. This increased efficacy would result in saving $0.93 million a year and also generate 15,000 minutes of extra procedure time for hospitals that annually perform about 5000 procedures. The time saved can be rightfully utilized for additional procedures. In addition, SSIs have been estimated to increase hospital expenses per admission by $ 20,842 [17]. So reduction of both extra procedure time and incidence of SSI, can lead to reduction in financial burden of the patient.

It is estimated that a large percentage of surgical site infections (up to 60%) can be prevented following proper surgical site preparation techniques [18]. Therefore, any preoperative skin cleaning technique whose efficacy is at least equal to that of the existing methods, but which is less dependent on user skill is likely to improve overall outcomes.

## Materials and methods

### Overview

**Figure 1:**
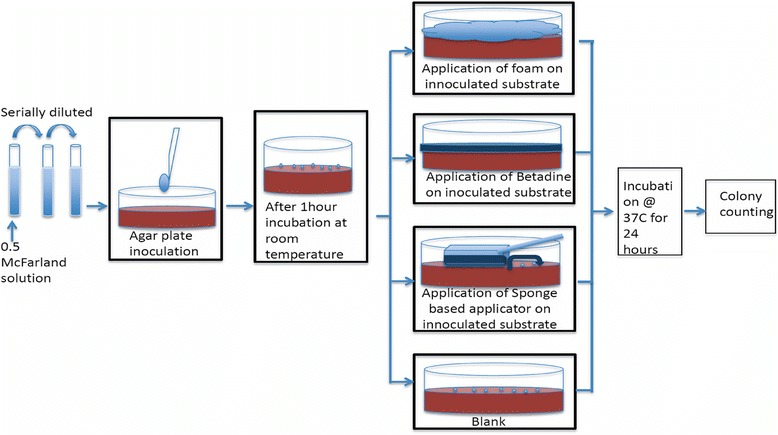
**Schematic of the method of testing the bactericidal efficacy.** This schematic details the methodology of testing the bactericidal efficacy using Povidone-iodine aerosol foam, sponge based applicator and flood-coverage with liquid Betadine.

The bactericidal efficacy of our spray-on formulation (povidone-iodine aerosol foam), is compared to that of the traditional sponge-based application method using the methodology schematically depicted in Figure 1. Details regarding individual steps in the protocol are described below. Standard 0.5 McFarland bacterial suspensions are spread on two different substrates: sterile nutrient agar and sterilized porcine skin. The substrates are incubated for an hour to allow sufficient time for the bacteria to adhere to the substrates. Post incubation, the surfaces are exposed to the various preoperative skin preparation techniques: povidone-iodine aerosol foam, wet skin scrub prep tray, flood-coverage with liquid Betadine (positive control) and flood-coverage with Phosphate buffer saline (PBS) or no-treatment (negative control). Post-exposure, the substrates are again incubated for 24 hours. After 24 hours, the number of surviving bacteria is estimated using colony counts, to estimate the bactericidal efficacy of the various techniques. 48 hours incubation of the plates is also done to validate if the bacteria are killed during the process or are merely injured and could grow if proper nutrients are provided to it [19].

### Bacterial cell culture

The micro-organisms used in the study are gram-positive bacteria *Staphylococcus aureus* ATCC 29213 and *Staphylococcus epidermidis* ATCC 12228 and gram-negative bacteria *Pseudomonas aeruginosa* ATCC 27853. The bacterial strains chosen for the study contribute to about 42% of SSI [7]. The strains are sub-cultured in Tryptic Soy Broth (TSB) and incubated for 20–24 hours at 37°C to obtain log-culture. Following which standard 0.5 McFarland bacterial suspension is prepared in TSB for use in the study using standard protocols [20].

### Selection of substrates

The antimicrobial efficacy of the agents used for preoperative skin preparation is tested *in vitro* using both agar plates as well as porcine skin. Tryptic Soy Agar (TSA) plates are used for culturing the bacteria.

Porcine skin is chosen for its close resemblance to human skin, and has previously also been used for studying the effect of chemicals on human skin [21,22]. Porcine skin purchased from local grocery shops is cleaned, de-haired and cut into one square inch pieces and sterilized, prior to use. The sterilization protocol, described in detail elsewhere [23] involved treating the skin pieces with an aqueous solution of 1 M sodium chloride and 0.1% (v/v) per-acetic acid with continuous stirring of the solution at 225 rpm for 30mins. It is followed by two times rinsing in PBS for a period of 24 hours each time by continuous stirring at 225 rpm [23,24].

### Selection of antimicrobial agents for testing

Liquid 5% Betadine is used for flood-coverage of the substrates. Preoperative wet skin scrub prep kit containing sponge applicators are used for the study with the applicators wetted with 5% Betadine prior to scrubbing. Aerosolized foam of povidone-iodine, 5% Betadine is used. The product is designed to be sprayed on the surgical site from a distance of 4–8 inches, resulting in the site being covered with foam. The propellant gases present in the aerosol foam canister mix with anti-microbial agents like povidone and iodine to produce a layer of foam. It requires less manual labor unlike scrubbing or painting. After predetermined contact time, the foam can be removed from the site with a sterile cloth thus making the site ready to be operated on.

In addition, to prove that there is no mechanical washing away of the bacteria on being flooded with 5% Betadine, the experiment was repeated with 1X PBS instead of 5% Betadine.

### Evaluation of bactericidal activity

For each bacterial culture, both agar plates and porcine skin are used. 5 agar plates are used for each case: control, flood-coverage by 5% Betadine, sponge-based applicator and povidone-iodine aerosolized foam (our product). Similarly, for porcine skin, a group of 5 one inch square skin pieces are used for each case, control, flood-coverage with 5% Betadine, sponge based applicator and our product.

Each bacterial culture is diluted to ~1*10^4^ CFU/ml and 100 μl is of the diluted bacteria is used for inoculation. It is thus expected that there will be ~10^3^ CFU on agar plates and porcine skin prior to disinfection. Post-inoculation, the agar plates and the porcine skin are allowed to incubate at room temperature for 1 hour. After this incubation time, the control samples of agar plates are allowed to incubate at 37°C for 24 hours. Imprint of the control samples of the porcine skin are taken on agar plates with the plates being incubated at 37°C for 24 hours. It may be noted that samples were incubated for 48 hours in the hope of detecting bacteria that were only injured, but not killed by the disinfecting techniques [19].

**Figure 2:**
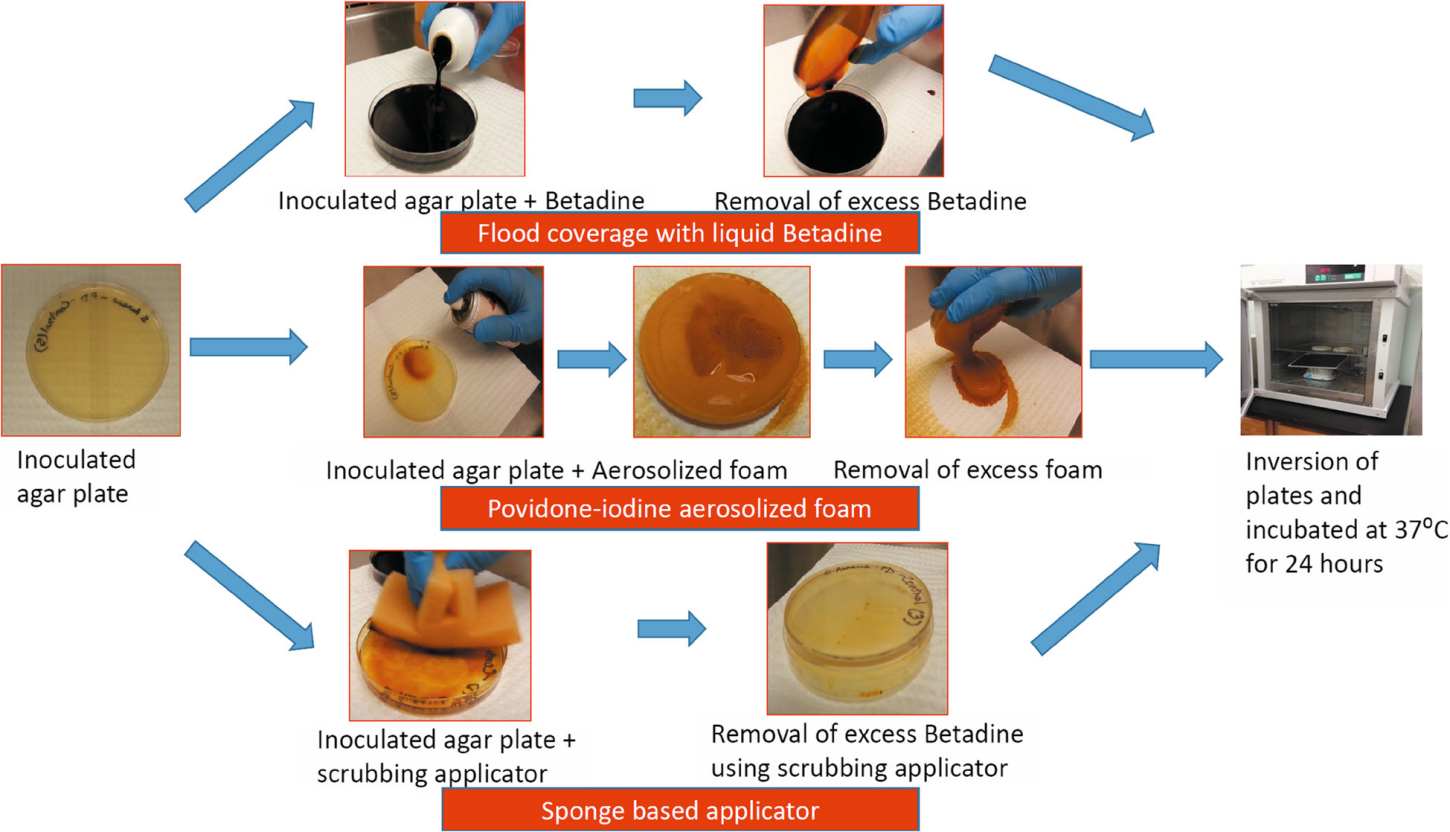
**Steps involved in evaluating bactericidal activity on nutrient agar plates.** Steps involved in treating the agar plates with flood-coverage of Betadine, aerosolized foam and sponge based applicator and evaluating its bactericidal activity.

**Figure 3:**
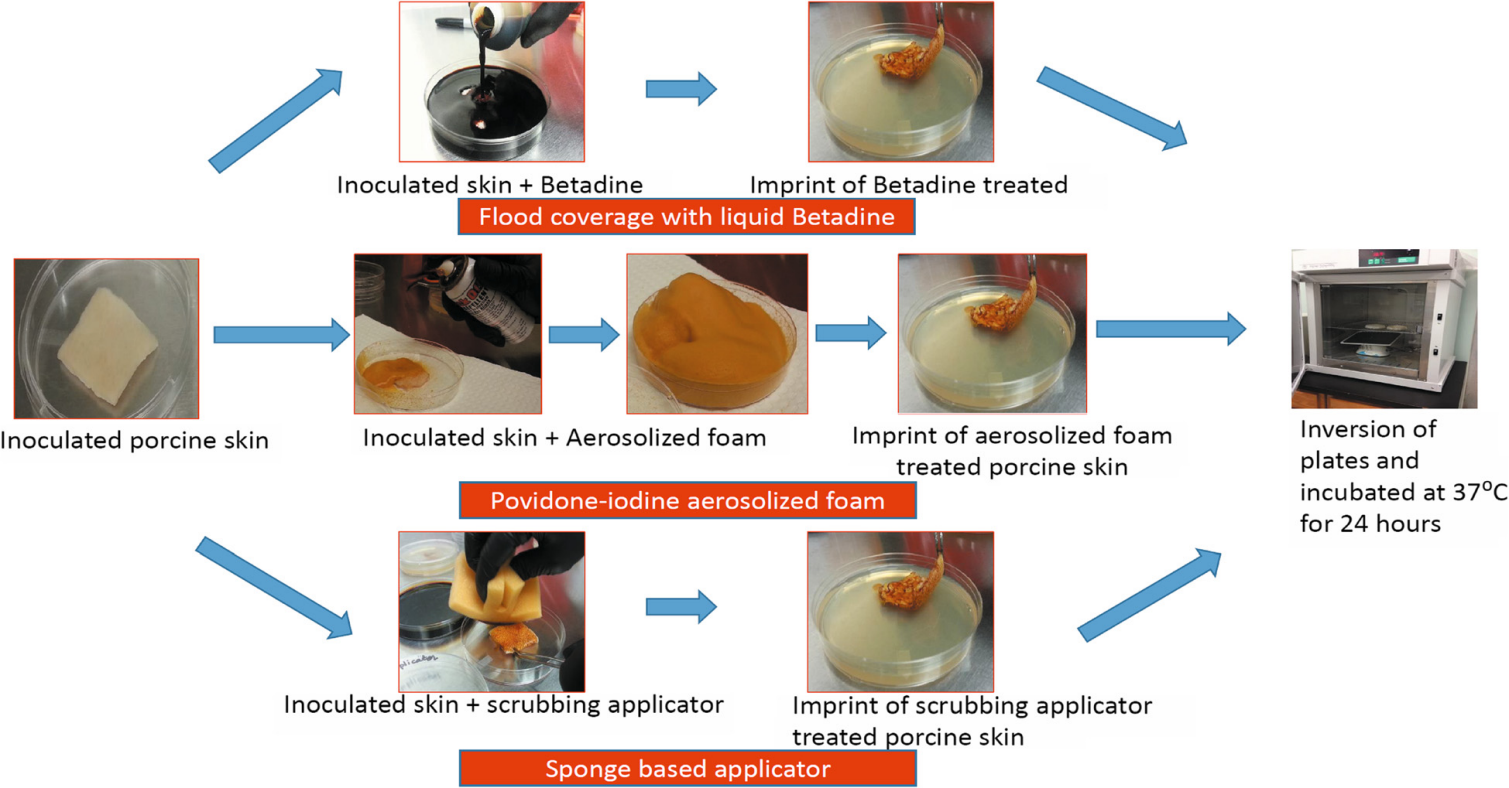
**Steps involved in evaluating bactericidal activity on porcine skin.** Steps involved in treating the porcine skin with flood-coverage of Betadine, aerosolized foam and sponge based applicator and evaluating its bactericidal activity.

The steps involved in treating the agar plates and the porcine skin with flood-coverage of Betadine, aerosolized foam and sponge based applicator are depicted in Figures 2 and 3, respectively.

For agar plates and skin pieces serving as substrate for 5% Betadine about 5-10 ml of liquid 5% Betadine, is poured on the surface. This ensures flooding and complete coverage of the entire surface of the agar plates/porcine skin with 5% Betadine. After 2 minutes of contact, the agar plates are flipped upside down to remove excess solution. In case of porcine skin, the skin is taken out and shaken well to remove excess solution and imprinted on agar plates.

For groups of agar plates and skin pieces being used for our product, aerosolized foam is sprayed on the surface. After two minutes of contact time the aerosolized foam is removed from the surface and in case of porcine skin the imprints of the skin are taken on agar plates. Similarly for the agar plates and porcine skin pieces used for sponge based applicator, the sponge post wetting with liquid 5% Betadine is gently rubbed on the inoculated surface in regular motion. The porcine skin is held firmly with a sterilized forceps to enable researchers to scrub the surface thoroughly, in a manner similar to the real world situation. Damaged plates resulting from scrubbing were discarded. Post surface rubbing with the wet sponge for two minutes, a dry sponge is used to remove the excess liquid. In case of the porcine skin, they are taken out and imprinted on agar plate.

### Statistical analysis

Statistical analysis was performed in Microsoft Excel using analysis of variance (ANOVA) single factor to establish the efficacy of the antimicrobial agents used. A measure of significance of differences is the p-value. The p-value thus provides a more intuitive feel of the degree of difference between the two quantities being compared. For our case, the null hypothesis is that the number of surviving bacteria (colonies) remaining is equal for the two processes that we compare, namely, aerosolized foam and sponge based applicator.

## Results

**Figure 4:**
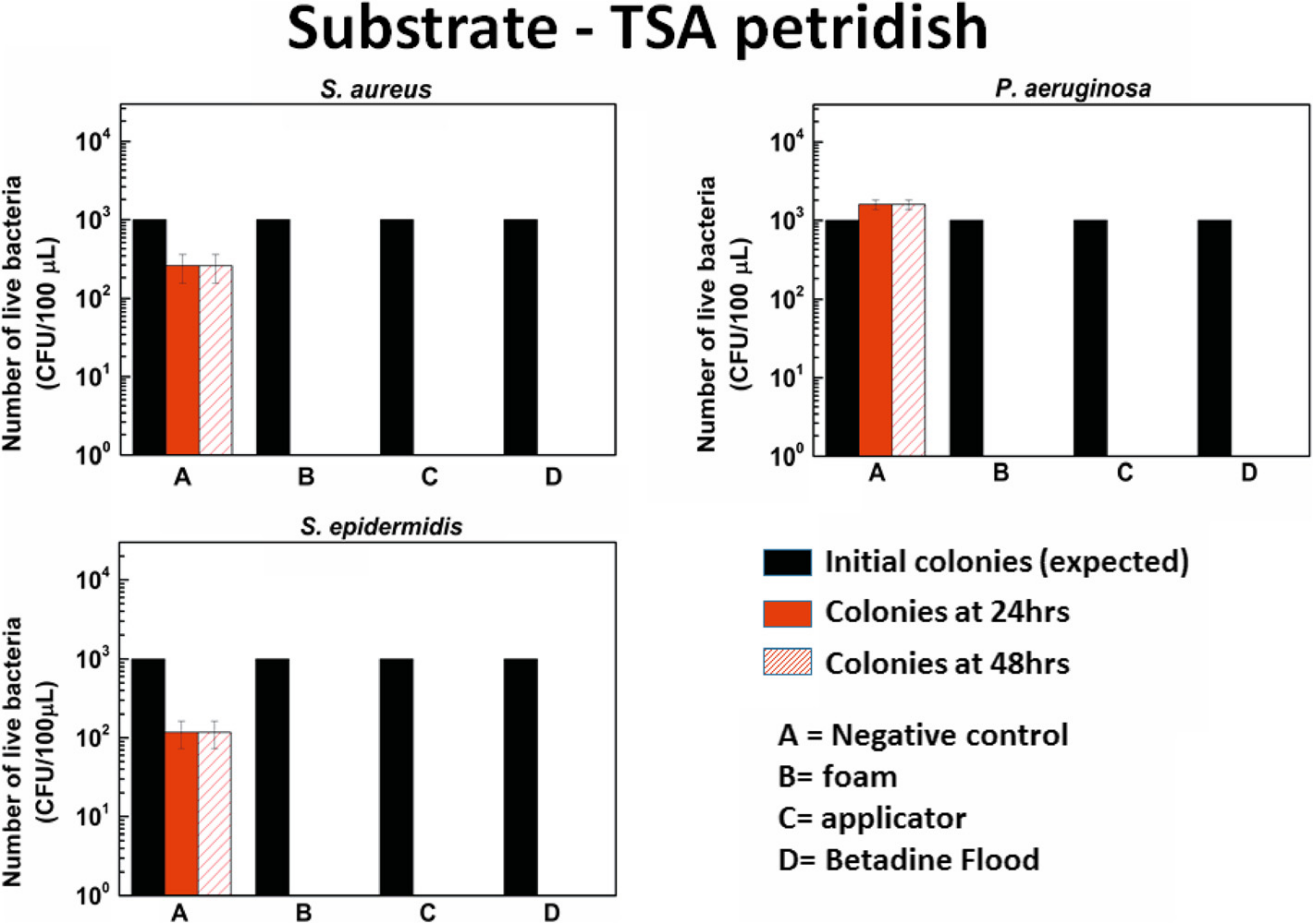
**Viable bacterial count for different bacteria tested on agar plates.** Number of live bacteria (CFU/100 μL) observed on agar plate after 24 hours and 48 hours for negative control (no treatment done), application of aerosolized foam, application of sponge based applicator and positive control (application of 5% Betadine) for *S. aureus* (top left) [n = 4], *P. aeruginosa* (top right) [n = 5] and *S. epidermidis* (bottom left) [n = 5].

Figure 4 shows the number of live bacteria remaining on the surface of the agar plate after addition of 100 μl suspension with 10^4^ CFU of bacteria/ml on a TSA petri-dish, and subsequent treatment with the disinfection protocols of interest. For the control (no disinfectant applied), this number starts at 1000 CFU and increases over 24 and 48 hours. More importantly, all the disinfection protocols, like povidone-iodine aerosol foam, sponge based applicator, and flood-coverage with liquid 5% Betadine (positive control) are equally effective for all three bacterial species tested (*S. aureus*, *P. aeruginosa* and *S. epidermidis*). All bacteria are eliminated (no colonies are seen after 24 or 48 hrs of incubation, post-treatment).

**Figure 5:**
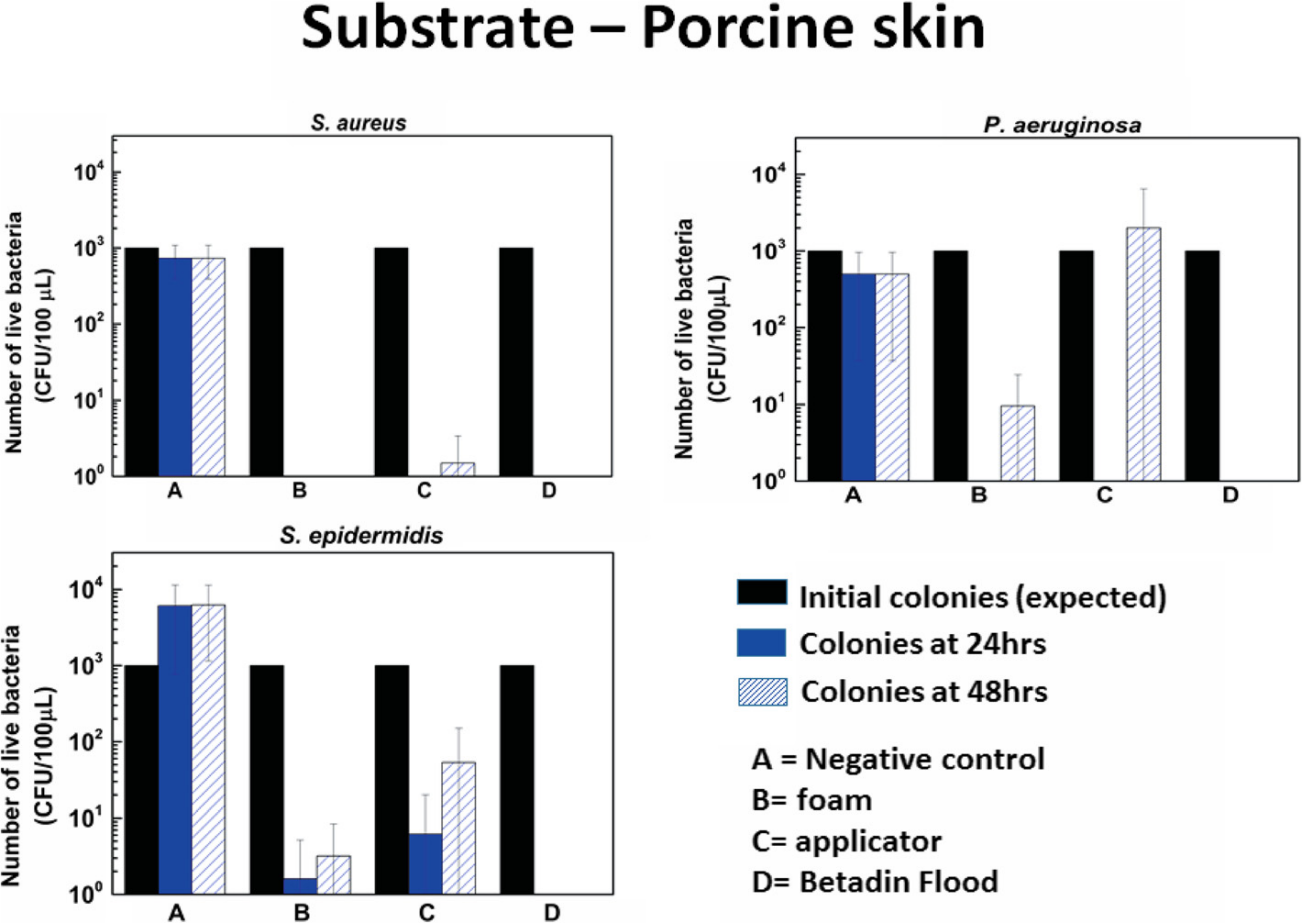
**Viable bacterial count for different bacteria tested on porcine skin.** Number of live bacteria (CFU/100 μL) observed on porcine skin after 24 hours and 48 hours for negative control (no treatment done), application of aerosolized foam, application of sponge based applicator and positive control (application of 5% Betadine) for *S. aureus* (top left) [n = 4], *P. aeruginosa* (top right) [n = 5] and *S. epidermidis* ((bottom left) [n = 5].

Figure 5 show the results of the tests conducted on porcine skin subjected to the same preoperative skin preparation (disinfection) techniques tested against the same set of bacteria. In this case, different disinfection protocols have different outcomes. It is noteworthy that (as expected for a positive control), the flood coverage with liquid Betadine is able to completely eliminate all bacteria present in this case as well. For the applicator sponge (currently used method for pre-operative skin disinfection), none of the bacteria tested were completely eliminated, although in 2 of the 3 cases (*S. aureus* and *P. aeruginosa*) all were at least injured (no colonies seen at 24 hrs). With the foaming betadine suspension (new product being investigated), the results obtained did not show statistically significant difference when compared to the sponge-applicator for all the bacteria tested. *S. aureus* was completely eliminated, and the number of colonies observed for the two other bacteria was lesser than the corresponding number for the sponge-applicator at both 24 and 48 hrs. The p-values obtained when comparing corresponding readings (same bacteria, same duration of incubation) are shown in Table 1.

**Table 1:** **p-values calculated using null hypothesis for all applications**

**Bacterial species**	**Observation time period**	**Porcine skin**
***S. aureus***	24 hrs	-
	48 hrs	0.1682
***P. aeruginosa***	24 hrs	0.4876
	48 hrs	0.3486
***S. epidermidis***	24 hrs	0.4929
	48 hrs	0.2819

List of p-values, as calculated for the null hypothesis that the two disinfection protocols (aerosolized foam and sponge based applicator) are equally effective. (n = 4 for *S. aureus*; n = 5 for *S. epidermidis* and *P. aeruginosa*).

When the experiment was repeated by flood coverage of the plates with 1X PBS it was observed that the plates showed bacterial growth similar to that of the control samples for both the substrates. This clearly indicated that there was no mechanical washing of the bacteria when flooded with Betadine but antimicrobial effects arose due to the chemical activity of 5% Betadine.

## Discussion

For agar plates as a substrate, the aerosolized foam, like the other existing methods of application, has a very strong bactericidal effect, as observed from the lack of colonies even after 48 hours for all the disinfection methods examined. Given that these plates provide a smooth surface for the antimicrobial agents to come into contact with the bacteria, it can be considered as an ideal case.

Porcine skin can be considered as a more realistic model for studying human skin in-vitro. Here, the bactericidal effect of the agents tested is mitigated by pores, folds and other structures in the skin that prevent the antimicrobial agents from sustained contact with the bacteria. Though all agents show approximately 2-log reductions in bacterial number, the tested foam did not have any statistically significant difference in efficacy when compared with the other agent/method tested as seen from the statistical analysis. It may be noted that none of the current surgical site preparation practices can completely sterilize skin.

Further, this is a preliminary study that merely looks at the efficacy of the foam against three bacterial species in-vitro, and it only suggests that the foam can be used for surgical site preparation. Additional studies on live animals and/or humans in a “real-world” setting may be needed before one can accurately establish how effective it is compared to other formulations/ methods available in the market, and whether there are any conditions under which it may not be as effective.

## Conclusion

This study shows that using a new delivery system (aerosolized foam) for an established antiseptic (betadine) achieved bactericidal results comparable to a traditional system that is currently used when tested in-vitro on both agar plates and porcine skin.

Given the limitations of this preliminary study, further studies venturing into clinical trials will be needed to obtain substantial evidence for SSI prevention using this product. Such studies will also explore how the aerosolized foam compares with many other kinds of surgical skin preparation products/techniques and whether the anticipated advantages of using the aerosolized foam (lower “error” rate due to its ease of use, and saving healthcare costs by virtue of requiring less time compared to the standard method) can be realized in a “real world” clinical setting.
